# Evaluation of Fly Ash Composition from Municipal Solid Waste Incinerators: The Role of the Incinerator Type and Flue Gas Deacidification Process

**DOI:** 10.3390/toxics13070588

**Published:** 2025-07-14

**Authors:** Xuetong Qu, Yanan Wang, Feifei Chen, Chuqiao Li, Yunfei He, Jibo Dou, Shuai Zhang, Jiafeng Ding, Hangjun Zhang, Yuchi Zhong

**Affiliations:** 1 School of Engineering, Hangzhou Normal University, Hangzhou 310018, China; 20240079@hznu.edu.cn (X.Q.); 2024111032017@stu.hznu.edu.cn (Y.W.); 2023111010085@stu.hznu.edu.cn (C.L.); heyyff@163.com (Y.H.); doujibo@hznu.edu.cn (J.D.); zhangsh33@163.com (S.Z.); djf101@hznu.edu.cn (J.D.); 20080099@hznu.edu.cn (H.Z.); 2 Zhejiang Provincial Key Laboratory of Wetland Intelligent Monitoring and Ecological Restoration, Hangzhou 311121, China; 3 Hangzhou Fuyang Huilong Environmental Protection Technology Co., Ltd., Hangzhou 330183, China; 13777592514@163.com

**Keywords:** MSWIs, fly ash, incinerator type, flue gas deacidification, oxide analysis, heavy metal, risk assessment

## Abstract

The resource utilization potential and environmental impact of fly ash from municipal solid waste incinerators (MSWIs) have attracted wide attention. In this study, four MSWIs in Hangzhou, Zhejiang Province were selected to systematically evaluate the effects of different incinerator types and flue gas deacidification processes on fly ash’s oxide and heavy metal components and their temporal changes as well as conduct risk assessment. The results showed that the contents of MgO, Al_2_O_3_, SiO_2_, and Fe_2_O_3_ in the grate furnace fly ash were significantly lower than those in the fluidized bed fly ash, but the compressive strength of its fly ash was high. Chemicals added during the flue gas deacidification process such as CaO and NaHCO_3_ significantly affected the contents of CaO and Na_2_O. In addition, heavy metals such as Cu, Mn, Cr, and Ni were mainly distributed in the fluidized bed fly ash, while heavy metals such as Pb and Cd were mainly collected in the grate furnace fly ash. The concentrations of various components in the fly ash fluctuated but were not significant under different time dimensions. Risk assessment indicated that heavy metals such as Cd, Pb, and Sb posed a high risk. This study is expected to provide theoretical support for the safe management and resource utilization of fly ash.

## 1. Introduction

With the acceleration of urbanization, the production of municipal solid waste (MSW) is on the rise. Currently, the global production of MSW is 2.01 billion tons per year, and this is expected to increase to 3.4 billion tons by 2050 [1] if a circular economy does not develop. The treatment of solid waste has become an urgent environmental problem that needs to be solved worldwide. Waste incineration technology has become a mainstream treatment method, with its significant volume reduction effect and resource utilization advantages [2,3,4]. As of 2023, China has built 696 municipal solid waste incinerators (MSWIs), and the scale of the industry continues to expand [5]. However, with the widespread application of waste incineration power generation technology, a large amount of incineration residue is also produced, including bottom ash and fly ash. While bottom ash constitutes the majority of the residue by volume, fly ash is of particular concern due to its fine particle size and high levels of toxic substances.

Oxides such as CaO, SiO_2_, Al_2_O_3_, and Fe_2_O_3_ in fly ash belong to the CaO-SiO_2_-Al_2_O_3_-Fe_2_O_3_ system and can be used as building materials and geotechnical engineering materials [6,7,8,9]. MgO, present in fly ash, is also advantageous for cement preparation [10]. In addition, the NMC/T value, calculated based on the oxide content, can be used as an essential indicator to predict the compressive strength of fly ash resource products, reflecting their engineering applicability [11]. However, fly ash is also rich in toxic heavy metals such as Pb, Hg, and Cd, which pose extremely high environmental risks. If improperly disposed of, then it may cause soil and groundwater pollution [12]. Therefore, the effective treatment and resource utilization of fly ash have become essential research directions for current waste incineration technology.

Different types of incinerators (such as grate furnaces and fluidized beds) and flue gas deacidification processes (such as dry, semi-dry, and sodium bicarbonate dry) affect the combustion temperature, residence time, and removal efficiency of pollutants in the flue gas, thereby changing the composition, heavy metal forms, and concentration of fly ash [13,14,15]. Fan et al. compared and evaluated the physical and chemical properties as well as the heavy metal concentrations of fly ash from a fluidized bed and a grate furnace. They found that the contents of Si, Al, and Fe in the fluidized bed fly ash were significantly higher than those in the grate furnace fly ash, while the levels of Pb, Cd, and Zn were lower [16]. Although previous studies have explored the impact of individual factors on fly ash characteristics [17], the combined effects of different incinerator types and flue gas deacidification processes on the fly ash composition, especially the dynamic changes of fly ash pollutants over time, require further investigation. In addition, there is still a lack of systematic comparative analysis of the risk levels of heavy metals in fly ash under different incinerators and flue gas deacidification processes.

This study takes four MSWIs in Hangzhou, Zhejiang Province as the research object, evaluates the effects of different furnace types and flue gas deacidification processes on fly ash composition, analyzes the dynamic changes of oxides and heavy metals in fly ash in different time dimensions, and conducts health and ecological risk assessment based on composition characteristics. This study aims to reveal the correlation mechanism between the incineration process, flue gas deacidification technology, and fly ash composition and provide theoretical support for optimizing waste incineration technology and formulating effective fly ash management strategies, thereby reducing environmental pollution and improving waste treatment efficiency.

## 2. Materials and Methods

### 2.1. Sample Collection

**Figure 1:**
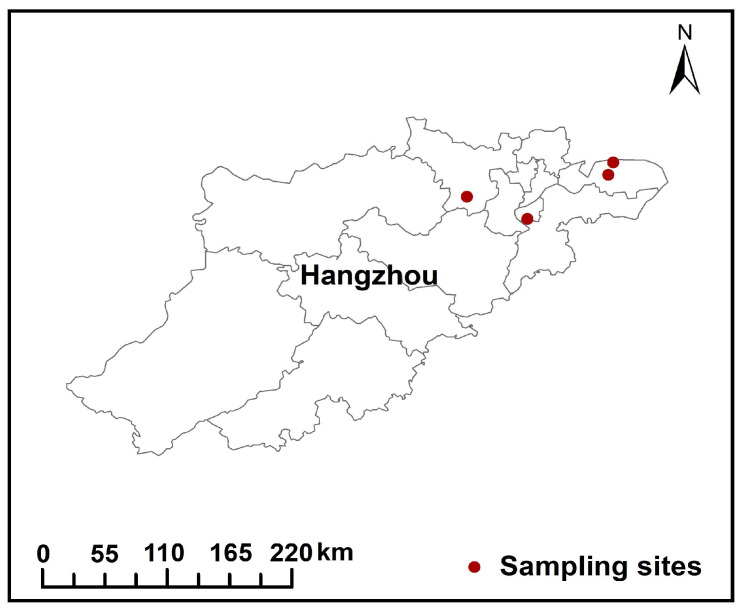
Locations of MSWIs in Hangzhou.

Four MSWIs in Hangzhou were selected to collect fly ash samples (Figure 1). The basic information of the four MSWIs is summarized in Table 1. MSWI-1 employs a fluidized bed incineration process and a dry and semi-dry deacidification process. MSWI-2 utilizes a grate furnace incineration process with sodium bicarbonate dry injection. MSWI-3 and MSWI-4 use a grate furnace incineration process and a dry and semi-dry deacidification process.

From January to December 2022, fly ash samples were systematically collected from the four MSWIs monthly. Five samples were randomly selected from each MSWI every month, resulting in a total of 240 samples. After collection, the samples were immediately sealed in polyethylene bags and transported to the laboratory for pretreatment and instrumental analysis.

### 2.2. Chemical Composition Analysis

The fly ash sample was dried at 105 °C for 8 h until a constant weight was achieved, after which it was stored for subsequent use. The composition of the fly ash was analyzed using an X-ray fluorescence spectrometer (XRF, Thermo Fisher, Waltham, MA, USA), with Rh as the target material, an excitation voltage of 60 kV, and an excitation current of 140 mA. NMC/T is calculated based on the oxide content, and the corresponding calculation equations are presented in Formula (1):

(1) $$\frac{\mathrm{NMC}}{T}=\frac{({\mathrm{Na}}_{2}O+{\;K}_{2}O+\mathrm{CaO}+\mathrm{MgO})}{(\mathrm{Si}O_{2}+{\mathrm{Al}}_{2}O_{3}+{\mathrm{Fe}}_{2}O_{3})}$$

### 2.3. Heavy Metal Analysis

The concentrations of seven heavy metals (Cu, Zn, Ni, Pb, Cd, Cr, and Mn) present in the fly ash samples were quantified using an inductively coupled plasma optical emission spectrometer (ICP-OES; Thermo Fisher Scientific, Waltham, MA, USA, iCAP 6300 Duo). In summary, approximately 10 g of fly ash was first freeze-dried and passed through a sieve with 150 µm openings. Fly ash with larger particle sizes was then finely ground and passed through the same sieve with 150 µm openings. From this, a 0.5 g aliquot was weighed and subjected to acid digestion using 9 mL HNO_3_ (Sinopharm Chemical Reagent Co., Ltd., Shanghai, China), 2 mL HCl (Sinopharm Chemical Reagent Co., Ltd., Shanghai, China), 3 mL HF (Sinopharm Chemical Reagent Co., Ltd., Shanghai, China), and 1 mL H_2_O_2_ (Sinopharm Chemical Reagent Co., Ltd., Shanghai, China). The mixture was then heated to facilitate decomposition. Subsequently, 2 mL HClO_4_ (Sinopharm Chemical Reagent Co., Ltd., Shanghai, China) was added, and the digestion continued until a dry residue was obtained. This residue was redissolved in 1% HNO_3_ and diluted to a fixed volume for analysis.

The concentrations of five heavy metals (As, Hg, V, Sb, and Sn) in fly ash were measured using an atomic fluorescence spectrometer (AFS; Beijing PuXi General Instrument Co., Beijing, China, AFS-9130). In brief, a 0.5 g portion of the sieved sample was accurately weighed, followed by the sequential addition of 6 mL HCl and 2 mL HNO_3_. The resulting mixture was subjected to a heating process to ensure complete digestion. After digestion, the solution was filtered, and the resulting filtrate was diluted to a predetermined volume for analysis.

Quality assurance and control procedures involved using blank samples, replicates, and standard reference materials. The detection limits for each targeted heavy metal are summarized in Table S1.

### 2.4. Statistical Analysis

Pearson correlation analysis was used to study the correlation between oxides and heavy metals in fly ash, and principal component analysis (PCA) was used to study the similarities and differences between the oxides and heavy metals in the fly ash. Before performing PCA, all compositional data were standardized (z-scores). PCA was carried out without the application of any rotation technique, such as Varimax, as the first two principal components accounted for a substantial proportion of the total variance. The analysis was performed using OriginPro 2021 (OriginLab Corporation, Northampton, MA, USA). The eigenvalues of the principal components are summarized in Table S2.

### 2.5. Risk Assessment

Based on the health risk assessment framework proposed by the United States Environmental Protection Agency (USEPA), both the carcinogenic and non-carcinogenic risks associated with heavy metals in fly ash were assessed. The evaluation was conducted through three exposure pathways: daily intake of chemicals (CDI_ing_), daily inhalation (CDI_inh_), and daily skin contact (CDI_der_). The corresponding calculation equations are presented in Formulas (2)–(4):

(2) $${\mathrm{CDI}}_{\mathrm{ing}}=\frac{C_{s}\;\times \;\mathrm{IngR}\;\times \;\mathrm{EF}\;\times \;\mathrm{ED}}{\mathrm{AT}\;\times \;\mathrm{BW}}\;\times \;10^{-6}$$

(3) $${\mathrm{CDI}}_{\mathrm{inh}}=\frac{C_{s\;}\times \;\mathrm{InhR}\;\times \;\mathrm{EF}\;\times \;\mathrm{ED}}{\mathrm{PEF}\;\times \;\mathrm{AT}\;\times \;\mathrm{BW}}$$

(4) $${\mathrm{CDI}}_{\mathrm{der}}=\frac{\;C_{s}\;\times \;\mathrm{SA}\;\times \;\mathrm{AF}\;\times \;\mathrm{ABS}\;\times \;\mathrm{EF}\;\times \;\mathrm{ED}}{\mathrm{AT}\;\times \;\mathrm{BW}}\;\times \;10^{-6}$$

Cs denotes the concentration of heavy metals in the fly ash (mg/kg). IngR represents the ingestion rate of fly ash particles (mg/day), while InhR refers to the inhalation rate of air (m^3^/day). EF is the exposure frequency (days/year), and ED corresponds to the exposure duration (year). AT stands for the average exposure time (day). PEF indicates the particulate emission factor (m^3^/kg). SA denotes the exposed skin surface area (cm^2^). AF is the skin adhesion factor (mg/cm^2^), and ABS represents the skin absorption factor (unitless). BW is the individual’s body weight (kg). The related parameters are shown in Table S3.

The carcinogenic risk (CR) of heavy metals was calculated using Formula (5):

(5) $${\mathrm{CR}}_{\mathrm{Fly}\;\mathrm{ash}}=\sum_{i=1}^{3}{\mathrm{CDI}}_{i}\;\times {\;\mathrm{SF}}_{i}$$

In this context, CR denotes the total carcinogenic risk resulting from three exposure pathways, SFi refers to the slope factor for carcinogenicity (mg/kg/day), and i indicates the three exposure pathways.

Non-carcinogenic risk was evaluated using the hazard index (HI), which was calculated using Formula (6):

(6) $${\mathrm{HI}}_{\mathrm{Fly}\;\mathrm{ash}}=\sum_{i=1}^{3}\frac{{\mathrm{CDI}}_{i}}{{\mathrm{RfD}}_{i}}$$

HI indicates the overall non-carcinogenic risk, and RfD refers to the reference dose (mg/kg/day) corresponding to the three exposure pathways. The related parameters are shown in Table S4.

The ecological risk of heavy metals was assessed using the risk index (RI), which was calculated as follows:

(7) $${\mathrm{RI}}_{\mathrm{Fly}\;\mathrm{ash}}=\sum E_{r}=\sum R_{i}\times \frac{C_{i}}{B_{i}}$$

Er represents the individual ecological risk factor for each heavy metal, and Ri denotes the metal’s toxic response factor [18,19]. Ci is the concentration of the heavy metal, and Bi refers to its corresponding background concentration in the environment. According to the Er value, the potential ecological risks can be divided into five categories. The threshold Er values for low, moderate, considerable, high, and very high ecological risks are Er < 40, 40 ≤ Er < 80, 80 ≤ Er < 160, 160 ≤ Er < 320, and ≥320, respectively. According to the RI value, the potential ecological risks can be divided into four categories. The threshold RI values for low, moderate, considerable, and high ecological risks are RI ≤ 150, 150 ≤ RI < 300, 300 ≤ RI < 600, and RI ≥ 600, respectively (Table S5).

## 3. Results

### 3.1. Oxide Analysis

**Figure 2:**
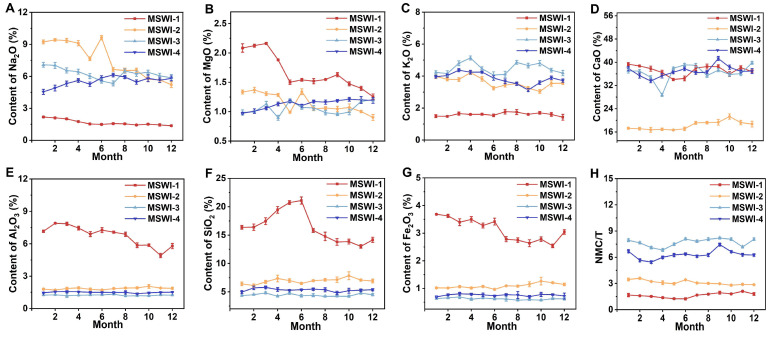
Changes of (**A**) Na_2_O, (**B**) MgO, (**C**) K_2_O, (**D**) CaO, (**E**) Al_2_O_3_, (**F**) SiO_2_, (**G**) Fe_2_O_3_, and (**H**) NMC/T in fly ash over time under different processes.

Figure 2 shows the dynamic effect of the incinerator type and flue gas deacidification on the oxide content in fly ash. The results showed that the fly ash differences between the fluidized bed and dry and semi-dry deacidification processes were the most significant ones. The contents of oxides such as MgO, Al_2_O_3_, SiO_2_, and Fe_2_O_3_ were significantly higher than those of other type of fly ash. It is worth noting that the contents of Na_2_O and K_2_O were significantly lower than those of other types of fly ash. Further comparison of the flue gas deacidification processes revealed that the sodium bicarbonate dry injection process had a lower CaO content but a higher Na_2_O content. Considering the temporal dimension, no statistically significant overall difference was observed (*p* > 0.05), although the oxide concentrations fluctuated with occasional short-term increases or decreases.

The NMC/T value indicates the compressive strength of the fly ash. The results show that the fly ash produced by the grate furnace and dry and semi-dry deacidification process had the better compressive strength, followed by the grate furnace and sodium bicarbonate dry injection process. In contrast, the compressive strength of the fly ash produced by the fluidized bed, combined with a dry and semi-dry deacidification process, was relatively low. The compressive strength of the fly ash in different processes did not change significantly over time (*p* > 0.05).

### 3.2. Heavy Metal Analysis

**Figure 3:**
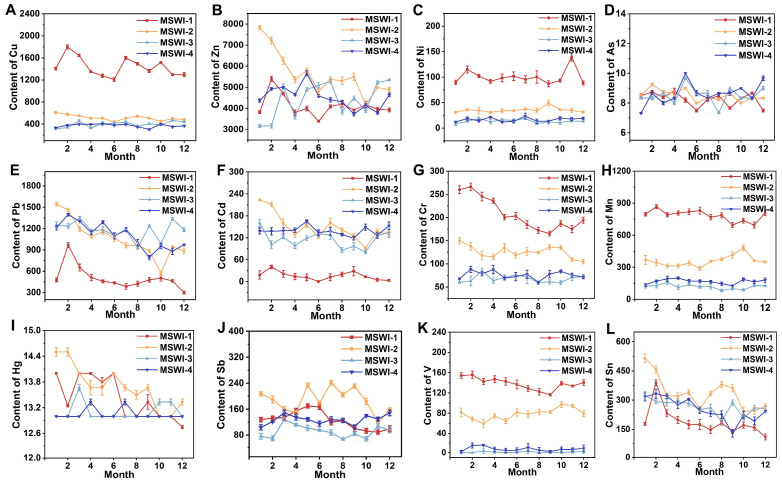
Changes of (**A**) Cu, (**B**) Zn, (**C**) Ni, (**D**) AS, (**E**) Pb, (**F**) Cd, (**G**) Cr, (**H**) Mn, (**I**) Hg, (**J**) Sb, (**K**) V, and (**L**) Sn in fly ash over time under different processes.

The effects of the incinerator type and flue gas deacidification process on the content of heavy metals in fly ash are shown in Figure 3. Similar to the effect of oxides, the fly ash of the fluidized bed with the dry and semi-dry deacidification process was most different from the other three processes. The contents of Cu, Ni, Cr, Mn, and V were significantly higher than those of other processes, but the contents of Pb and Cd were significantly lower than those of the other three processes.

The sodium bicarbonate dry injection process resulted in higher concentrations of Ni, Cr, Mn, Sb, V, and Sn compared with conventional dry and semi-dry flue gas deacidification methods. Among the heavy metals, the contents of As and Hg were less affected by the process, and there was no noticeable difference. Compared with oxides, the contents of heavy metals in fly ash fluctuate greatly over time.

### 3.3. Correlation Analysis

**Figure 4:**
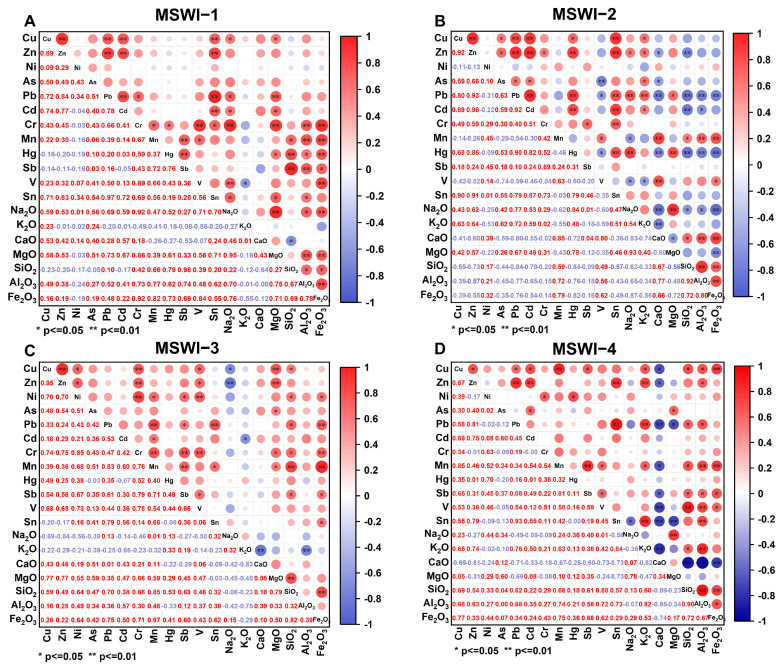
Correlation analysis of oxides and heavy metals in fly ash from four MSWIs: (**A**) MSWI-1, (**B**) MSWI-2, (**C**) MSWI-3, (**D**) MSWI-4. * *p* < 0.05. ** *p* < 0.01.

The relationships between oxides and heavy metals in fly ash under different incineration process conditions were analyzed (Figure 4, Tables S6–S9). The results showed significant differences between oxides and heavy metals under different incineration conditions. In MSWI-1 (fluidized bed with dry and semi-dry treatment), oxides and heavy metals were, for the most part, significantly positively correlated (Figure 4A). For example, Cr, Mn, Hg, and Sb and SiO_2_, Al_2_O_3_, and Fe_2_O_3_ were all significantly positively correlated. In addition, Cr and Na_2_O were also significantly correlated. For negative correlation, there was only one group: V and K_2_O. In MSWI-2 (grate furnace with sodium bicarbonate dry treatment), the proportion of significant negative correlation between oxides and heavy metals increased significantly (Figure 4B). Pb and Hg and MgO, SiO_2_, Al_2_O_3_, and Fe_2_O_3_ were all significantly negatively correlated. However, Mn was still significantly positively correlated with MgO, SiO_2_, Al_2_O_3_, and Fe_2_O_3_. MSWI-3 and MSWI-4 are both grate furnaces with dry and semi-dry processes, and their correlation spectra were similar overall, with only individual compounds showing differences (Figure 4C,D). To further reveal the effects of the incinerator type and deacidification process on the composition of MSWI fly ash, PCA was performed (Figure S1). The first two principal components explained 82.2% of the total variance (PC1: 61.9%; PC2: 20.3%) (Table S2). The results showed that, based on the oxide and heavy metal data, the fly ash samples from different types of incinerators and deacidification processes exhibited a clear separation trend.

### 3.4. Health and Ecological Risk Assessment

**Figure 5:**
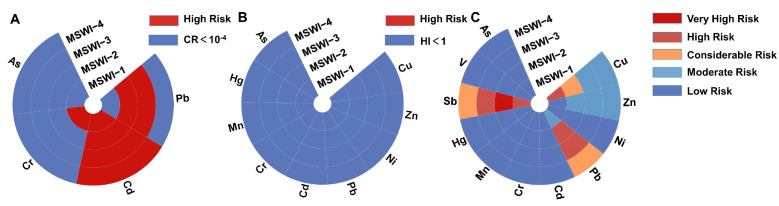
(**A**) Carcinogenic risk, (**B**) non-carcinogenic risk, and (**C**) ecological risk of heavy metals in fly ash from four MSWIs.

The health and ecological risks of heavy metals in fly ash from the four MSWIs are shown in Figure 5. Tables S10–S12 show the CR_Fly ash_, HI_Fly ash_, and RI_Fly ash_ values of the heavy metals. Health risks can be divided into carcinogenic risks and non-carcinogenic risks. The carcinogenic risks of heavy metals in the fly ash from the four MSWIs exceeded the acceptable level (CR < 10^−4^), among which MSWI-4 had the highest CR_Fly ash_ value of 0.001468. Cd, which as the heavy metal with the highest carcinogenic risk should be strictly controlled (Figure 5A). For non-carcinogenic risks, the levels of individual heavy metals were within the acceptable limits (HI < 1) (Figure 5B). However, the HI_Fly ash_ value was not ideal. Except for MSWI-1, which remained within acceptable levels, the other MSWIs exceeded the acceptable threshold, with MSWI-2 showing the highest risk at 1.23 (Table S11). Heavy metals in fly ash will migrate to the ecosystem after leaching, thus bringing potential ecological risks. The heavy metals in fly ash from the four MSWIs showed serious ecological risks, especially MSWI-2, which showed extremely high ecological risks. For MSWI-1, Cu and Sb were the main-risk heavy metals, while the other three should focus on Pb and Sb (Figure 5C). The difference in risk among heavy metals also shows that the type of incinerator has a greater impact on the fly ash composition. It is worth noting that the fly ash produced by the grate furnace and sodium bicarbonate dry process has the highest health and ecological risk.

## 4. Discussion

At present, grate furnaces and fluidized bed incinerators are the two common types of incinerators in MSWIs. Previous studies have shown that under the same treatment conditions, the MSWI fly ash produced by grate furnaces per unit of waste treatment is usually less than that of fluidized bed incinerators. This is primarily due to the differences between the two types of furnaces in terms of combustion temperature, residence time, and mixing efficiency, which significantly affect the amount of fly ash generated, as well as its particle size distribution and composition characteristics. Our study reveals the effect of the incinerator type on the distribution of oxide concentrations in fly ash. During fluidized bed incineration, the flue gas is rich in fine particles such as aluminum silicate and calcium magnesium iron oxide [20]. These aerosol particles are effectively captured in the bag filter and deacidification reactor and enrich the fly ash, thereby increasing the content of oxides such as MgO and SiO_2_ in the fly ash. However, due to the large number of flue gas particles in the fluidized bed, the fly ash produced during the deacidification process is diluted, resulting in a decrease in the contents of Na_2_O and K_2_O in the fly ash [21]. During the incineration of MSW, the flue gas deacidification process will also significantly affect the contents of oxides in fly ash. The dry, semi-dry, and sodium bicarbonate dry injection processes all involve spraying chemicals (such as lime powder or sodium bicarbonate) into the flue gas to remove pollutants through chemical reactions. However, the type of chemical used impacts the oxide composition in the fly ash. For example, in the sodium bicarbonate dry injection process, the injected NaHCO_3_ will increase the content of Na_2_O in the fly ash, while the traditional dry and semi-dry processes usually use Ca(OH)_2_ or CaO as a deacidification agent, which significantly increases the proportion of CaO in the fly ash and affects the content distribution of other oxides.

In addition to the influence on the distribution of oxides in fly ash, different incinerator types and flue gas deacidification processes also have an important influence on the mechanical properties of fly ash. The NMC/T value can effectively indicate the compressive strength of fly ash, providing a scientific basis for the resource utilization and safe disposal of fly ash [22]. A grate furnace and dry and semi-dry deacidification processes can enhance the compressive strength of fly ash, broadening its potential applications in building materials. Notably, the compressive strength of fly ash under different process conditions remains relatively stable over time (*p* > 0.05), indicating that it has good mechanical and chemical stability under these conditions.

The contents of heavy metals in fly ash are also affected by the incinerator type and flue gas deacidification process. Fluidized bed incinerators usually operate at higher temperatures and have a strong gas-solid mixing effect and uniform temperature distribution [23]. Under these conditions for the fluidized bed, some heavy metals that are typically less volatile (such as Mn, Cu, Cr, Ni, and V) can also be carried by the flue gas and become enriched in the fly ash particles [24]. In contrast, the higher chlorine content in the grate furnace can significantly enhance the volatility of Pb and Cd, which then condense on the fly ash particles during the cooling process, increasing their concentrations in the fly ash [25]. The sodium bicarbonate dry spraying process reacts with acidic gases to form salts and may react with some heavy metals, promoting the enrichment of elements such as Ni, Cr, Mn, Sb, V, and Sn in fly ash [26]. As and Hg are highly volatile and easily enter the flue gas in gaseous form at high temperatures. They are not easily adsorbed or captured, resulting in a slight difference in the contents of these elements in fly ash [27]. Compared with the oxide content, the volatility of heavy metal contents in fly ash is more obvious, which is mainly attributed to the dynamic change characteristics of the incineration process itself [28]. Parameters such as the incinerator temperature, atmosphere composition, and reaction time will change during operation, thus affecting the morphological transformation and migration behavior of heavy metals. In contrast, metal oxides usually exist in a stable, solid form, and their formation mechanism is less affected by changes in incineration conditions. Therefore, their content in fly ash is relatively stable.

The results of the correlation analysis revealed the potential relationship between oxides and heavy metal elements in fly ash. In the dry and semi-dry deacidification process, heavy metals such as Cr, Mn, Hg, and Sb are significantly positively correlated with oxides such as SiO_2_, Al_2_O_3_, and Fe_2_O_3_. This positive correlation can be explained by the following mechanism. Both Hg and Sb can be effectively fixed by SiO_2_, Al_2_O_3_, and Fe_2_O_3_ through chemical adsorption, complexation, and co-precipitation mechanisms, resulting in a significant positive correlation between their contents and these oxides [29,30]. In addition, heavy metals such as Cr and Mn may also be embedded in the lattice of SiO_2_, Al_2_O_3_, or Fe_2_O_3_ [31,32]. For the sodium bicarbonate dry injection process, NaHCO_3_ reacts with gasified heavy metals to form carbonates. However, the affinity between carbonates and silicon aluminum oxides (SiO_2_ and Al_2_O_3_) is low, resulting in a negative correlation between some heavy metals (Pb and Hg) and oxides [33,34]. However, under high-temperature incineration, Mn easily forms manganese silicates or aluminates with SiO_2_ and Al_2_O_3_ and therefore is positively correlated with these oxides [35]. It is worth noting that there is a significant negative correlation between V and K_2_O because V prefers to combine with acidic substances, while K_2_O is an alkaline oxide [36]. PCA analysis shows that the concentrations of heavy metals and oxides in fly ash can indicate different furnace types and incineration processes well.

The risk assessment results show that heavy metals in different MSWI fly ashes have potential health and ecological risks, among which the risk level of the MSWI-2 samples is the highest and requires special attention. Among the many heavy metals, Cd, Sb, Pb, Cu, and Zn are the main risk contributors and should be the key control targets for fly ash risk management and disposal. Overall, the research results emphasize that in practical applications, it is necessary to pay attention to the influence of the process conditions on the occurrence characteristics and risk levels of heavy metals in fly ash, optimize the incineration process and flue gas treatment measures, ensure that effective pollution prevention and control measures are taken, and reduce the enrichment and release of high-risk heavy metals in fly ash from the source to reduce its potential environmental and health hazards.

## 5. Conclusions

This study evaluated the effects of different incinerator types and flue gas deacidification processes on the contents of oxides and heavy metals in fly ash from MSWIs. The study showed that the incinerator type significantly affected the composition of the fly ash. Fluidized bed fly ash contained high levels of oxides such as MgO, Al_2_O_3_, SiO_2_, and Fe_2_O_3_ and heavy metals such as Cu, Mn, Cr, and Ni, but it had a lower compressive strength than the grate furnace fly ash. The effect of the flue gas deacidification process on fly ash oxides was mainly reflected in the contents of CaO and Na_2_O. The contents of the heavy metals Cr, Mn, and V in the fly ash were higher under the sodium bicarbonate dry injection process. The correlation heat map revealed the potential correlation between fly ash oxides and heavy metals under different incinerator types and flue gas deacidification processes. Risk assessment showed that heavy metals in fly ash have potential health and ecological risks. The results of this study provide a scientific basis for the selection of furnace types and the optimization of flue gas deacidification processes in MSWIs. They also provide a feasible solution for environmental protection treatment and the resource utilization of fly ash.

## Figures and Tables

**Table 1:** The basic information of the four MSWIs.

Sample No.	Incinerator Type	Flue Gas Deacidification	Capacity (t/d)	Operating Hours (h)
MSWI-1	Fluidized bed	Dry and semi-dry	600	82,080
MSWI-2	Grate furnace	Sodium bicarbonate dry	150	178,560
MSWI-3	Grate furnace	Dry and semi-dry	870	38,880
MSWI-4	Grate furnace	Dry and semi-dry	750	65,520

## Data Availability

The data supporting the findings of this study are included within the paper and Supplementary Materials and are available from the corresponding authors on request.

## Supplementary Materials

The following supporting information can be downloaded at https://www.mdpi.com/article/10.3390/toxics13070588/s1. Table S1: Method performance parameters for heavy metal determination. Table S2: The eigenvalues of the principal components. Table S3: Health risk parameters of heavy metals. Table S4: Slope factors (SFs) and reference doses (RfDs) of heavy metals in fly ash from different exposure pathways. Table S5: Indicators and classification of potential ecological risk for metal pollution. Table S6: The *p* value of MSWI-1. Table S7: The *p* value of MSWI-2. Table S8: The *p* value of MSWI-3. Table S9: The *p* value of MSWI-4. Table S10: Carcinogenic risk of heavy metals in fly ash from different MSWIs. Table S11: Non-carcinogenic risk of heavy metals in fly ash from different MSWIs. Table S12: Ecological risks of heavy metals in fly ash from different MSWIs. Figure S1: Principal component analysis between different MSWIs.

## References

[1] Kaza S., Yao L.C., Bhada-Tata P., Woerden F.V. What a Waste 2.0: A Global Snapshot of Solid Waste Management to 2050. Urban Development Series Washington, D.C.: World Bank Group. http://documents.worldbank.org/curated/en/697271544470229584.

[2] Vukovic N., Makogon E. (2022). Waste-to-Energy Generation: Complex Efficiency Analysis of Modern Technologies. Sustainability.

[3] Nanda S., Berruti F. (2021). A technical review of bioenergy and resource recovery from municipal solid waste. J. Hazard. Mater..

[4] Zhang Y., Wang L., Chen L., Ma B., Zhang Y., Ni W., Tsang D.C.W. (2021). Treatment of municipal solid waste incineration fly ash: State-of-the-art technologies and future perspectives. J. Hazard. Mater..

[5] (2023). National Bureau of Statistics of China. https://data.stats.gov.cn/easyquery.htm?cn=C01&zb=A0C06&sj=2023.

[6] Ghouleh Z., Shao Y. (2018). Turning municipal solid waste incineration into a cleaner cement production. J. Clean. Prod..

[7] Yan D., Peng Z., Yu L., Sun Y., Yong R., Helge Karstensen K. (2018). Characterization of heavy metals and PCDD/Fs from water-washing pretreatment and a cement kiln co-processing municipal solid waste incinerator fly ash. Waste Manag..

[8] Sarmiento L.M., Clavier K.A., Paris J.M., Ferraro C.C., Townsend T.G. (2019). Critical examination of recycled municipal solid waste incineration ash as a mineral source for portland cement manufacture—A case study. Resour. Conserv. Recycl..

[9] Medici F., Piga L., Rinaldi G. (2000). Behaviour of polyaminophenolic additives in the granulation of lime and fly-ash. Waste Manag..

[10] Wu K., Shi H., Schutter G.D., Guo X., Ye G. (2012). Preparation of alinite cement from municipal solid waste incineration fly ash. Cem. Concr. Compos..

[11] Moon G.D., Oh S., Choi Y.C. (2016). Effects of the physicochemical properties of fly ash on the compressive strength of high-volume fly ash mortar. Constr. Build. Mater..

[12] Lou Y., Jiang S., Du B., Dai X., Wang T., Wang J., Zhang Y. (2023). Leaching morphology characteristics and environmental risk assessment of 13 hazardous trace elements from municipal solid waste incineration fly ash. Fuel.

[13] Hwang I.-H., Matsuo T., Matsuto T., Tojo Y., Sameshima R. (2021). Dry scrubbing of municipal solid waste incineration flue gas using porous sodium carbonate produced via vacuum thermal treatment of sodium bicarbonate. J. Mater. Cycles Waste Manag..

[14] Ma X., He T., Da Y., Su F., Yang R. (2024). The Toxicity Leaching and the Cement Admixtures Properties with Incineration Fly Ash of Different Furnace Types. Langmuir.

[15] Ning H., Tang R., Li C., Gu X., Gong Z., Zhu C., Li J., Wang K., Yu J. (2025). Recent advances in process and materials for dry desulfurization of industrial flue gas: An overview. Sep. Purif. Technol..

[16] Fan C., Wang B., Ai H., Liu Z. (2022). A comparative study on characteristics and leaching toxicity of fluidized bed and grate furnace MSWI fly ash. J. Environ. Manag..

[17] Wang W., Tian S., Long J., Liu J., Ma Q., Xu K., Zhang Z. (2022). Investigation and Evaluation of Flue Gas Pollutants Emission in Waste-to-Energy Plant with Flue Gas Recirculation. Atmosphere.

[18] Hakanson L. (1980). An ecological risk index for aquatic pollution control. A sedimentological approach. Water Res..

[19] Zhao W., Ding L., Gu X., Luo J., Liu Y., Guo L., Shi Y., Huang T., Cheng S. (2015). Levels and ecological risk assessment of metals in soils from a typical e-waste recycling region in southeast China. Ecotoxicology.

[20] Liu Z., Li J.B., Zhu M.M., Cheng F.Q., Lu X.F., Zhang Z.Z., Zhang D.K. (2020). An experimental investigation into the effect of flue gas recirculation on ash deposition and Na migration behaviour in circulating fluidized bed during combustion of high sodium Zhundong lignite. Fuel Process. Technol..

[21] Yang S., Song G., Na Y., Yang Z. (2018). Alkali metal transformation and ash deposition performance of high alkali content Zhundong coal and its gasification fly ash under circulating fluidized bed combustion. Appl. Therm. Eng..

[22] Cho Y.K., Jung S.H., Choi Y.C. (2019). Effects of chemical composition of fly ash on compressive strength of fly ash cement mortar. Constr. Build. Mater..

[23] Lin K., Zhao Y., Kuo J.H., Lin C.L. (2023). Agglomeration-influenced transformation of heavy metals in gas-solid phases during simulated sewage sludge co-incineration: Effects of phosphorus and operating temperature. Sci. Total Environ..

[24] Zhang S., Jiang X., Liu B., Lv G., Jin Y., Yan J. (2017). Co-combustion of Bituminous Coal and Pickling Sludge in a Drop-Tube Furnace: Thermodynamic Study and Experimental Data on the Distribution of Cr, Ni, Mn, As, Cu, Sb, Pb, Cd, Zn, and Sn. Energy Fuels.

[25] Liu Z., Yue Y., Lu M., Zhang J., Sun F., Huang X., Zhou J., Qian G. (2019). Comprehension of heavy metal stability in municipal solid waste incineration fly ash with its compositional variety: A quick prediction case of leaching potential. Waste Manag..

[26] Kalisz S., Wejkowski R., Maj I., Garbacz P. (2023). A novel approach to the dry desulfurization process by means of sodium bicarbonate: A full-scale study on SO_2_ emission and geochemistry of fly ash. Energy.

[27] Zhao S., Duan Y., Lu J., Liu S., Pudasainee D., Gupta R., Liu M., Lu J. (2018). Enrichment characteristics, thermal stability and volatility of hazardous trace elements in fly ash from a coal-fired power plant. Fuel.

[28] Hailu S.L., McCrindle R.I., Seopela M.P., Combrinck S. (2019). Speciation of major and trace elements leached from coal fly ash and the kinetics involved. J. Environ. Sci. Health.

[29] Marczak G.M., Piersa P., Karczewski M., Szufa S., Ünyay H., Kędzierska S.A., Bochenek P. (2021). Modified Fly Ash-Based Adsorbents (MFA) for Mercury and Carbon Dioxide Removal from Coal-Fired Flue Gases. Energies.

[30] Zhou C.C., Liu G.J., Xu Z.Y., Sun H., Kwan Sing Lam P. (2018). Retention mechanisms of ash compositions on toxic elements (Sb, Se and Pb) during fluidized bed combustion. Fuel.

[31] Mao L.Q., Deng N., Liu L., Cui H., Zhang W.Y. (2016). Effects of Al_2_O_3_, Fe_2_O_3_, and SiO_2_ on Cr(VI) formation during heating of solid waste containing Cr(III). Chem. Eng. J..

[32] Xing Y.Q., Wang B.M. (2024). Chemical speciation, distribution, and leaching behaviors of heavy metals in alkali-activated converter steel slag-based stabilization/solidification of MSWI FA. Constr. Build. Mater..

[33] Li Y.K., Feng D.D., Bai C.X., Sun S.Z., Zhang Y., Zhao Y.J., Li Y.Z., Zhang F., Chang G.Z., Qin Y.K. (2022). Thermal synergistic treatment of municipal solid waste incineration (MSWI) fly ash and fluxing agent in specific situation: Melting characteristics, leaching characteristics of heavy metals. Fuel Process. Technol..

[34] Qin J., Zhang Y., Yi Y., Fang M. (2022). Carbonation treatment of gasification fly ash from municipal solid waste using sodium carbonate and sodium bicarbonate solutions. Environ. Pollut..

[35] Wang J., Cao C., Zhang Y., Su Z., Jiang T. (2022). Influence of Al_2_O_3_-induced MnO_2_–SiO_2_ smelting on silicate phase and consolidation behavior of manganese ore sinters. Ceram. Int..

[36] Gustafsson J.P. (2019). Vanadium geochemistry in the biogeosphere–speciation, solid-solution interactions, and ecotoxicity. Appl. Geochem..

