# Supplementary material for: Evaluation of Fly Ash Composition from Municipal Solid Waste Incinerators: The Role of the Incinerator Type and Flue Gas Deacidification Process
Source: Toxics. 2025 Jul 14;13(7):588. doi: 10.3390/toxics13070588 (PMC12300090; doi:10.3390/toxics13070588)
Supplement: Supplementary file 1 [file toxics-13-00588-s001.zip › toxics-3713102-supplementary.pdf]

Supporting Information

Evaluation of Fly Ash Composition from Municipal Solid Waste Incinerators: The Role of Incinerator Type and Flue Gas Deacidification Process

Xuetong Qu[1,2*], Yanan Wang[1,2], Feifei Chen[3], Chuqiao Li[1,2], Yunfei He[1,2], Jibo Dou[1,2], Shuai Zhang[1,2], Yunfei He[1,2], Jiafeng Ding [1,2], Hangjun Zhang[1,2], Yuchi Zhong[3*]

1 School of Engineering, Hangzhou Normal University, 310018, Hangzhou, Zhejiang, China

2 Zhejiang Provincial Key Laboratory of Wetland Intelligent Monitoring and Ecological Restoration, 311121, Hangzhou, Zhejiang, China

3 Hangzhou Fuyang Huilong Environmental Protection Technology Co., LTD, 330183, Hangzhou, Zhejiang, China

Table S1. Method performance parameters for heavy metal determination.

Element	Detection Limit (mg/kg)	RSD (%)
Cu	0.40	2.90
Zn	1.20	3.10
Ni	0.40	7.70
Pb	1.40	3.50
Cd	0.10	7.10
Cr	0.50	5.20
Mn	3.10	4.40
Hg	0.002	8.50
Sb	0.01	6.60
V	1.50	7.80
Sn	0.40	4.10
As	0.30	1.50

Table S2. The eigenvalues of principal components

Principal Component Number	Eigenvalue	Percentage of Variance (%)	Cumulative (%)
1	11.7687	61.9408	61.9408
2	3.8476	20.2505	82.1913
3	1.2831	6.7531	88.9444
4	0.6893	3.6277	92.5721
5	0.4634	2.4391	95.0112
6	0.2959	1.5572	96.5684
7	0.2110	1.1106	97.6791
8	0.1450	0.7629	98.4420
9	0.0806	0.4239	98.8659
10	0.0639	0.3364	99.2023
11	0.0418	0.2200	99.4222
12	0.0347	0.1825	99.6048
13	0.0267	0.1403	99.7450
14	0.0203	0.1071	99.8521
15	0.0152	0.0798	99.9319
16	0.0052	0.0273	99.9592
17	0.0041	0.0218	99.9810
18	0.0020	0.0106	99.9915
19	0.0016	0.0085	100

Table S3. Health risk parameters of heavy metals.

Parameters	Adult	Definition/Units
C_s	-	Concentration (mg I-TEQ/kg)
InhR	20	Inhalation rate (m^3/day)
EF	350	Exposure frequency (day/year)
ED	53	Exposure duration (year)
PEF	1.36E+9	Particle emission factor (m^3/kg)
AT	Carcinogenic:365*70<br>Non-carcinogenic:365*53	Average exposure time (day)
BW	56.80	Body weight (kg)
IngR	100	Ingestion rate (mg/day)
SA	5700	Surface area of the skin (cm^2)
AF	0.07	Skin adherence factor (mg/cm^2)
ABS	0.001	Dermal absorption factor (Unitless)

Table S4. Slope factor (SF) and reference dose (RfD) of heavy metals in fly ash from different exposure pathways.

Heavy Metal	SF_i (mg/kg/day)			RfD_i (mg/kg/day)		
	SF_{inh}	SF_{ing}	SF_{der}	RfD_{inh}	RfD_{ing}	RfD_{der}
Cu	-	-	-	0.04	0.04	1.20E-03
Zn	-	-	-	0.30	0.30	0.06
Ni	-	-	-	2.06E-02	2.00E-02	8.00E-04
Pb	0.08	0.08	0.08	3.52E-03	3.50E-03	5.25E-04
Cd	1.80E-03	6.10	6.10	1.00E-03	1.00E-03	1.00E-05
Cr	4.20	0.50	2.00	2.68E-05	3.00E-03	6.00E-05
As	15.10	1.50	3.66	3.00E-04	3.00E-04	1.23E-04
Mn	-	-	-	1.43E-05	4.60E-02	1.84E-03
Hg	-	-	-	8.57E-05	2.00E-04	1.60E-04

Table S5. Indicators and classification of potential ecological risk for metal pollution.

E_r	RI	**Ecological risk level**
$E_r < 40$	$RI < 150$	Low
$40 \leq E_r < 80$	$150 \leq RI < 300$	Moderate
$80 \leq E_r < 160$	$300 \leq RI < 600$	Considerable
$160 \leq E_r < 320$	$RI \geq 600$	High
$320 \leq E_r$		Very High

Table S6. The p value of MSWI-1.

p Value	Cu	Zn	Ni	As	Pb	Cd	Cr	Mn	Hg	Sb	V	Sn	Na_2O	K_2O	CaO	MgO	SiO_2	Al_2O_3	Fe_2O_3
Cu																			
Zn	0.000																		
Ni	0.784	0.362																	
As	0.098	0.109	0.167																
Pb	0.008	0.001	0.287	0.089															
Cd	0.006	0.003	0.910	0.201	0.003														
Cr	0.163	0.142	0.929	0.159	0.019	0.184													
Mn	0.489	0.350	0.622	0.861	0.215	0.660	0.017												
Hg	0.584	0.537	0.558	0.758	0.541	0.935	0.044	0.234											
Sb	0.654	0.728	0.613	0.928	0.626	0.888	0.163	0.009	0.004										
V	0.482	0.307	0.827	0.184	0.095	0.691	0.000	0.019	0.166	0.246									
Sn	0.009	0.001	0.282	0.068	0.000	0.008	0.012	0.061	0.547	0.373	0.060								
Na_2O	0.044	0.074	0.987	0.057	0.013	0.044	0.000	0.123	0.082	0.381	0.009	0.012							
K_2O	0.452	0.988	0.979	0.419	0.545	0.982	0.115	0.185	0.589	0.817	0.055	0.545	0.425						
CaO	0.074	0.171	0.655	0.206	0.371	0.050	0.576	0.415	0.401	0.076	0.824	0.460	0.138	0.980					
MgO	0.048	0.077	0.917	0.088	0.006	0.016	0.000	0.208	0.033	0.298	0.057	0.010	0.000	0.600	0.169				
SiO_2	0.475	0.532	0.589	0.872	0.753	0.598	0.178	0.019	0.002	0.000	0.207	0.529	0.492	0.722	0.025	0.387			
Al_2O_3	0.106	0.219	0.458	0.397	0.080	0.178	0.007	0.003	0.032	0.006	0.116	0.030	0.010	0.988	0.997	0.005	0.017		
Fe_2O_3	0.632	0.549	0.552	0.561	0.120	0.499	0.000	0.001	0.007	0.013	0.001	0.065	0.004	0.070	0.709	0.009	0.014	0.003	

Table S7. The p value of MSWI-2.

p Value	Cu	Zn	Ni	As	Pb	Cd	Cr	Mn	Hg	Sb	V	Sn	Na_2O	K_2O	CaO	MgO	SiO_2	Al_2O_3	Fe_2O_3
Cu																			
Zn	0.000																		
Ni	0.731	0.692																	
As	0.044	0.019	0.746																
Pb	0.002	0.000	0.324	0.027															
Cd	0.000	0.000	0.483	0.043	0.000														
Cr	0.102	0.044	0.356	0.335	0.195	0.090													
Mn	0.670	0.423	0.139	0.386	0.067	0.337	0.178												
Hg	0.015	0.000	0.770	0.075	0.000	0.001	0.081	0.134											
Sb	0.568	0.456	0.141	0.581	0.758	0.446	0.012	0.445	0.333										
V	0.171	0.084	0.666	0.006	0.044	0.128	0.891	0.027	0.040	0.527									
Sn	0.000	0.000	0.973	0.062	0.002	0.000	0.007	0.915	0.002	0.130	0.258								
Na_2O	0.164	0.032	0.424	0.172	0.004	0.074	0.360	0.030	0.001	0.964	0.040	0.122							
K_2O	0.028	0.026	0.074	0.030	0.008	0.044	0.946	0.061	0.116	0.577	0.043	0.092	0.069						
CaO	0.181	0.041	0.208	0.042	0.002	0.065	0.957	0.000	0.008	0.892	0.002	0.224	0.001	0.006					
MgO	0.171	0.052	0.491	0.421	0.017	0.106	0.329	0.167	0.003	0.715	0.266	0.136	0.000	0.202	0.040				
SiO_2	0.062	0.007	0.607	0.156	0.001	0.002	0.523	0.042	0.001	0.790	0.109	0.051	0.030	0.287	0.018	0.058			
Al_2O_3	0.271	0.052	0.522	0.146	0.004	0.022	0.632	0.009	0.004	0.576	0.060	0.168	0.022	0.279	0.003	0.111	0.000		
Fe_2O_3	0.213	0.063	0.303	0.266	0.004	0.068	0.665	0.002	0.001	0.628	0.031	0.107	0.000	0.060	0.000	0.009	0.009	0.002	

Table S8. The p value of MSWI-3.

p Value	Cu	Zn	Ni	As	Pb	Cd	Cr	Mn	Hg	Sb	V	Sn	Na_2O	K_2O	CaO	MgO	SiO_2	Al_2O_3	Fe_2O_3
Cu																			
Zn	0.000																		
Ni	0.012	0.011																	
As	0.117	0.072	0.087																
Pb	0.299	0.455	0.164	0.173															
Cd	0.572	0.363	0.513	0.248	0.075														
Cr	0.006	0.005	0.000	0.166	0.119	0.179													
Mn	0.205	0.249	0.015	0.089	0.001	0.037	0.004												
Hg	0.108	0.424	0.224	0.934	0.270	0.826	0.086	0.195											
Sb	0.072	0.058	0.018	0.270	0.034	0.343	0.002	0.009	0.102										
V	0.014	0.015	0.007	0.682	0.155	0.247	0.003	0.067	0.148	0.021									
Sn	0.534	0.591	0.623	0.187	0.002	0.058	0.661	0.020	0.797	0.247	0.849								
Na_2O	0.013	0.001	0.060	0.207	0.697	0.658	0.131	0.984	0.676	0.395	0.096	0.304							
K_2O	0.483	0.356	0.505	0.214	0.443	0.019	0.473	0.310	0.296	0.553	0.657	0.479	0.306						
CaO	0.168	0.131	0.563	0.087	0.975	0.161	0.508	0.725	0.499	0.354	0.856	0.784	0.170	0.001					
MgO	0.003	0.004	0.066	0.045	0.265	0.128	0.016	0.045	0.365	0.144	0.127	0.929	0.137	0.144	0.062				
SiO_2	0.042	0.109	0.026	0.120	0.011	0.222	0.015	0.000	0.078	0.029	0.129	0.303	0.859	0.471	0.584	0.002			
Al_2O_3	0.610	0.384	0.104	0.286	0.249	0.052	0.345	0.134	0.291	0.700	0.236	0.210	0.177	0.005	0.215	0.300	0.316		
Fe_2O_3	0.384	0.498	0.025	0.170	0.005	0.099	0.010	0.000	0.181	0.038	0.166	0.032	0.646	0.358	0.768	0.101	0.001	0.206	

Table S9. The p value of MSWI-4.

p Value	Cu	Zn	Ni	As	Pb	Cd	Cr	Mn	Hg	Sb	V	Sn	Na_2O	K_2O	CaO	MgO	SiO_2	Al_2O_3	Fe_2O_3
Cu																			
Zn	0.016																		
Ni	0.205	0.606																	
As	0.343	0.203	0.956																
Pb	0.048	0.001	0.941	0.700															
Cd	0.013	0.005	0.801	0.041	0.143														
Cr	0.277	0.985	0.029	0.771	0.562	0.992													
Mn	0.000	0.129	0.080	0.450	0.278	0.071	0.068												
Hg	0.262	0.973	0.011	0.527	0.626	0.984	0.221	0.313											
Sb	0.022	0.326	0.144	0.243	0.799	0.102	0.493	0.001	0.724										
V	0.076	0.256	0.132	0.884	0.070	0.710	0.089	0.047	0.620	0.045									
Sn	0.064	0.002	0.782	0.696	0.000	0.063	0.733	0.174	0.996	0.551	0.145								
Na_2O	0.467	0.389	0.154	0.278	0.105	0.798	0.789	0.450	0.255	0.200	0.971	0.048							
K_2O	0.022	0.006	0.943	0.764	0.004	0.100	0.514	0.030	0.681	0.248	0.170	0.001	0.210						
CaO	0.013	0.035	0.452	0.712	0.010	0.294	0.572	0.016	0.424	0.039	0.007	0.007	0.837	0.001					
MgO	0.875	0.334	0.363	0.038	0.013	0.813	0.807	0.760	0.700	0.259	0.444	0.007	0.003	0.125	0.273				
SiO_2	0.012	0.070	0.299	0.903	0.030	0.486	0.361	0.015	0.586	0.035	0.000	0.055	0.682	0.038	0.000	0.464			
Al_2O_3	0.015	0.027	0.388	0.988	0.015	0.261	0.389	0.006	0.515	0.048	0.008	0.008	0.821	0.001	0.000	0.276	0.000		
Fe_2O_3	0.004	0.299	0.154	0.839	0.275	0.456	0.160	0.005	0.256	0.014	0.032	0.359	0.359	0.074	0.006	0.600	0.008	0.017	

Table S10. Carcinogenic risk of heavy metals in fly ash from different MSWIs.

	MSWI-1	MSWI-2	MSWI-3	MSWI-4
Pb	0.000045	0.000112	0.000133	0.000095
Cd	0.000219	0.001143	0.000877	0.001315
Cr	0.000165	0.000086	0.000051	0.000040
As	0.000019	0.000017	0.000019	0.000017
$CR_{Fly\ ash}$	0.000448	0.001359	0.001080	0.001468

Table S11. Non-carcinogenic risk of heavy metals in fly ash from different MSWIs.

	MSWI-1	MSWI-2	MSWI-3	MSWI-4
Cu	0.07	0.02	0.02	0.02
Zn	0.02	0.03	0.02	0.02
Ni	0.01	0.00	0.00	0.00
Pb	0.22	0.54	0.64	0.46
Cd	0.07	0.34	0.26	0.40
Cr	0.17	0.09	0.05	0.04
Mn	0.05	0.02	0.01	0.01
Hg	0.12	0.12	0.11	0.11
As	0.06	0.05	0.06	0.05
$HI_{Fly\ ash}$	0.78	1.23	1.18	1.11

Table S12. Ecological risks of heavy metals in fly ash from different MSWIs.

	MSWI-1	MSWI-2	MSWI-3	MSWI-4
Cu	225.97	82.31	62.82	52.92
Zn	39.85	59.29	43.71	41.40
Ni	13.89	4.78	2.62	1.85
Pb	72.20	179.77	212.83	152.96
Cd	5.53	28.82	22.11	33.16
Cr	6.55	3.43	2.04	1.57
Mn	1.31	0.61	0.25	0.20
Hg	3.31	3.31	3.08	3.08
Sb	206.78	362.71	213.56	133.90
V	2.96	1.17	0.32	0.00
As	13.18	11.86	13.18	11.86
$RI_{Fly\ ash}$	591.53	738.05	576.52	432.90

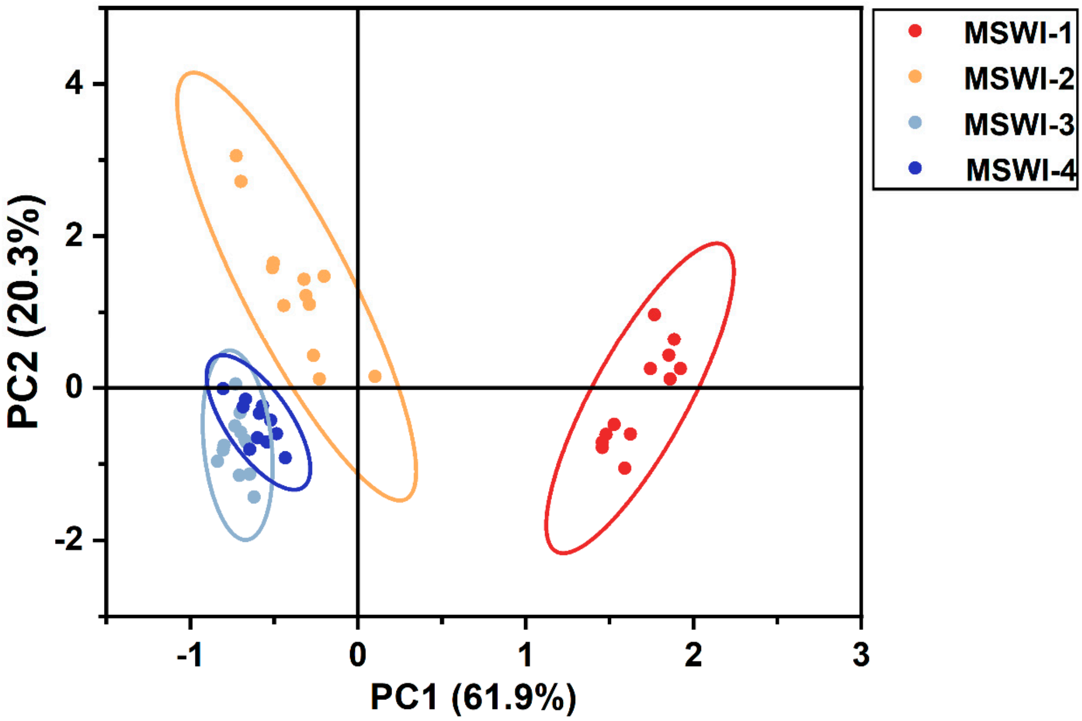

Figure S1. Principal component analysis between different MSWIs.
